# Paget's disease of the breast in male with underlying invasive ductal carcinoma: A case report with review of literature

**DOI:** 10.1016/j.amsu.2021.103035

**Published:** 2021-11-11

**Authors:** Abdulwahid M. Salih, Zuhair D. Hammood, Fahmi H. Kakamad, Snur Othman, Razhan K. Ali, Shaban Latif

**Affiliations:** a Department of Surgery, College of Medicine, University of Sulaimani, Sulaimani, Iraq; b Smart Health Tower, Madam Mittarand Str., Sulaimani, Iraq; c Kscien Organization, Hamdi Street, Azadi Mall, Sulaimani, Iraq; d Department of Cardiothoracic Surgery, Shar Hospital, Sulaimani, Iraq

**Keywords:** Paget's disease, Invasive ductal carcinoma, Male, Breast

## Abstract

**Introduction:**

Paget's disease of the breast is a rare cutaneous eczema-like condition that occurs in the nipple-areolar complex of the breast. The current study aims to report a rare case of Paget's disease of the breast associated with invasive ductal carcinoma in a male breast.

**Case report:**

A 54-year-old male presented with ulceration of the left nipple-areolar complex that has been progressing over the last 6 months. On examination; there was a palpable axillary lymph node. On ultrasound, a small hypoechoic heterogenous mass was seen beneath the areola (8*4 mm) with surrounding vascularity, and a few axillary lymph nodes with normal morphology and cortical thickness. The mammography revealed some points of calcification arranged in clusters. The patient underwent left side mastectomy and sentinel lymph node biopsy. The result of histopathological examination showed left side unifocal invasive ductal carcinoma.

**Discussion:**

There are two main theories that explain the histogenesis of Paget's disease of the breast with and without underlying malignancy: epidermotropic theory, which suggests an epidermal infiltration of the nipple with Paget cells, and transformative theory, which proposes a malignant transformation of normal glandular cells of the epidermis.

**Conclusion:**

The clinical presentation of Paget's disease of the breast is characteristic and should always warn the surgeon of the possibility of underlying malignancy.

## Highlights

• Paget's disease of the breast (or nipple) is a very rare cutaneous entity.
• Paget's disease of the male breast is an extremely rare clinicopathologic condition.
• Breast cancer in male is likewise very rare.

## 1 Introduction

Paget's disease of the breast is a rare cutaneous eczema-like condition that occurs in the nipple-areolar complex of the breast [1]. It makes up 1–3% of all primary breast cancers [2]. It was first described by James Paget as an ulceration of the nipple associated with an underlying breast cancer [3]. Previous studies revealed that nearly 82%–100% of PD patients have an associated underlying malignancy [4]. It usually occurs in conjunction with invasive ductal carcinoma (IDC) and ductal carcinoma in situ (DCIS) [5]. PD without associated malignancy of the breast parenchyma is called pTis [1]. Although there is a 10% increase in the incidence of both IDC and DCIS over the past few years, PD incidence has decreased by 45% during the same period of time [5]. PD of the male breast is an extremely rare clinicopathologic condition [6]. Breast cancer in male is likewise a rare condition accounting for nearly 1% of all breast cancers [7]. Compounded together, Paget's disease of the male breast with underlying invasive ductal carcinoma is an extremely rare condition.

The current study aims to report a rare case of PD of the breast associated with IDC in a male breast. The report has been arranged in line with SCARE 2020 guidelines with a brief literature review [8].

## 2 Case report

**Patient's information**: A 54-year-old male presented with ulceration of the left nipple-areolar complex that has been progressing over the last 6 months. He had no past medical or surgical history.

**Clinical examination**: The patient had ulceration of the left nipple-areolar complex and palpable axillary lymph nodes.

**Diagnostic assessment**: On ultrasound, a small hypoechoic heterogenous mass was seen beneath the nipple-areolar complex (8*4 mm) with surrounding vascularity, and a few axillary lymph nodes with normal morphology and cortical thickness. The mammography showed some points of calcification arranged in clusters (20*15mm). Core biopsy revealed high grade DCIS with no invasion. Wedge resection of the nipple and areola revealed Paget's disease of the nipple with dermal lymphatic permeation by tumor cell emboli. Fine needle aspiration cytology (FNAC) of the left axillary lymph node was negative for malignancy.

**Therapeutic intervention**: The patient underwent left side mastectomy and sentinel lymph node biopsy. The result of histopathological examination showed left side unifocal invasive ductal carcinoma, grade 2, moderately differentiated, 60% intermediate-high grade DCIS, cribriform and solid patterns with comedo necrosis within and outside of the invasive tumor along with nipple PD with dermal lymphatic permeation. Two lymph nodes were isolated and one of them was involved with micro-metastases without extra-nodal extension, pT1cN1m (sn).

**Follow up**: Post-operative period was uneventful. The patient left the hospital on the second post-operative day.

## 3 Discussion

Paget's disease is characterized by infiltration of the nipple and areolar epidermis by large tumor cells of glandular differentiation [9]. It accounts for 1.45% of all male breast malignancy and 0.68% of all female breast malignancies [10]. The underlying malignancy is usually of ductal type and has a high histologic grade [9]. The incidence of PD without an underlying malignancy is more prominent in males [7]. Several studies reported that only 10% of mammary PD is ER positive [11].

There are two main theories explaining the histogenesis of PD with and without underlying malignancy. The first one is the epidermotropic theory which suggests an epidermal infiltration of the nipple with Paget cells [12]. This is supported by the presence of underlying malignancy in the majority of cases, and the fact that the associated underlying malignancy and PD have the same immunohistochemical characteristic and gene expression patterns [3]. PD without an underlying malignancy can be explained by the second theory, transformative theory, which proposes that the disease is due to malignant transformation of normal glandular cells of the epidermis [13]. The current case is associated with unifocal IDC.

The identified risk factors include advanced age, genetic conditions such as Klinefelter's syndrome, conditions that may lead to estrogen and progesterone imbalance (infertility, obesity, and cirrhosis), benign diseases of the breast (breast cyst and trauma), and radiation exposure [6]. Up to 10% of men with positive BRCA2 mutation develop breast cancer [14]. Certain mutations found in female breast cancer (BRIP1, RAD51C) have not been found to be associated with male breast cancer [10]. The median age at presentation is 68 years in men but around 5 years younger in women [6].

The presenting case is 54 years old with negative past medical and family history. The most common presentations of PD include eczematous destruction of the nipple, crusting, scaling, bleeding, and ulceration [15]. The eczematous change nearly always starts on the nipple and then extending to the areola [16]. Palpable breast mass may be present in approximately 50% of cases [17]. The nipple changes may precede the enlargement of breast mass by many months [18]. However, PD can be found incidentally (histopathological examination of surgical specimen following mastectomy) without any gross nipple-areolar complex changes [19]. PD without an underlying malignancy is usually confined to the areola similarly in both sexes [20]. The disease is usually unilateral and the underlying malignancy can be located in any part of the breast with the majority being central [16,21]. However, bilateral PD of the breast have been reported in few reports [6]. Bansal et al. reported a rare presentation of PD with an extensive cutaneous involvement (involving the entire breast) without any associated malignancy [22]. In this report, the case presented with unilateral ulceration of the left nipple-areolar complex with a palpable ipsilateral axillary lymph node.

There are many conditions that may mimic PD of the breast such as psoriasis, contact dermatitis, erosive adenomatosis of the nipple, intraductal papilloma, nipple adenoma, basal cell carcinoma, Bowen's disease, malignant melanoma, and Toker cell hyperplasia [23]. It is commonly mistaken with benign dermatological diseases of the nipple, particularly, dermatitis [24]. PD of the breast can be distinguished from a locally advanced breast cancer presenting as satellite skin nodules [10].

Mammography should be recommended for all cases with clinical features of PD to detect any underlying malignancy and to follow up cases who undergo conservative treatment [3]. The features on mammography include skin thickening, malignant calcification, architectural distortion, and nipple retraction [25]. However, mammography may underestimate the true existence of underlying malignancy in up to 43% of PD [26]. When mammography is negative, ultrasound (US) may be helpful and can be considered as an alternative evaluation technique for detection of small masses [17]. Mammography of the current case revealed some points of calcifications, and the ultrasound revealed a small hypoechoic mass just beneath the nipple-areolar complex. MRI is a very useful and accurate diagnostic test for PD without a palpable breast mass and normal ultrasonography [27]. MRI has more sensitivity (95%) in detection of breast masses compared to mammography (70%) [28]. It is also useful to evaluate the extension of the tumor [25].

The duration from the onset of symptoms to treatment ranges from 12 to 14 months [12]. Misdiagnosis, treatment with topical steroid, and normal radiography are the most common causes of delay in the diagnosis and treatment [3].

Mastectomy with and without axillary lymph node dissection is the surgical procedure of choice for PD patients because of the high likelihood of association with multifocal or multicentric lesions of the breast parenchyma [29]. Breast conserving therapy followed by whole breast irradiation has emerged as a popular form of treatment modality in patients where the disease is in its earlier stage and there is no evidence of malignancy on clinical and radiological examination [22]. Moreover, some studies reported that the breast conserving therapy followed by whole breast radiotherapy is the treatment of choice for patients with both invasive and noninvasive carcinoma, and recommended that sentinel lymph node biopsy should be done even with normal clinical and radiological findings [16,29]. Mastectomy with sentinel lymph node biopsy was performed on the current reported case.

The prognosis of PD with associated malignancy depends on the characteristics and the staging of the underlying malignancy [7]. Chen et al. reported that PD may alter the prognosis of DCIS but not of IDC [4]. The prognosis of PD with an underlying malignancy differed significantly from the same breast cancer without PD [4].

In conclusion, the clinical presentation of Paget's disease of the breast is quite straightforward. It should always alert the surgeon of underlying malignancy which will alter the diagnostic and therapeutic approaches. This in turn will affect the overall prognosis.

## Consent

Consent has been taken from the patient and the family of the patient.

## Provenance and peer review

Not commissioned, externally peer reviewed.

## Conflicts of interest

There is no conflict to be declared.

## Sources of funding

No source to be stated.

## Ethical approval

Approval is not necessary for case report in our locality.

## Consent

Consent has been taken from the patient and the family of the patient.

## Author contribution

Abdulwahid M. Salih, Zuhair D. Hammood, Shaban Latif: surgeons diagnosing the case, follow up the patient, and final approval of the manuscript. Fahmi H. Kakamad, Snur Othman, Razhan K. Ali: literature review, writing the manuscript, final approval of the manuscript.

## Registration of research studies

Registration is not required for case report.

## Guarantor

Fahmi Hussein Kakamad is the Guarantor of submission.
